# Retraction: Targeted Knockdown of IQGAP1 Inhibits the Progression of Esophageal Squamous Cell Carcinoma *In Vitro* and *In Vivo*

**DOI:** 10.1371/journal.pone.0267634

**Published:** 2022-04-20

**Authors:** The PLOS ONE Editors

Following the publication of this article [1], concerns were raised regarding the methods and results. Specifically,

- In Fig 6, tumor volume appears to exceed 2000 mm^3^, and tumors appear ulcerated. The tumor data raise concerns about animal welfare and the adherence of the study to animal research ethics standards.
- In the Methods section, the sequence of the control siRNA used in the siRNA knockdown experiment was not provided.
- In Fig 5A, the KYSE150 control and K2 0 h panels appear to partially overlap.
- From the raw western blot image data provided during discussions, it appears that the following cropped panels did not originate from the same location on the membrane or were from different membranes:
  ○ Fig 2A: IQGAP1 and β-actin panels.
  ○ Fig 2C: IQGAP1 and β-actin panels for KYSE150.
  ○ Fig 4B: all panels.
  ○ S1A Fig: IQGAP1 and β-actin panels for EC9706 and KYSE150.

The corresponding author acknowledged that tumor volume was calculated incorrectly in the published figure, and they provided updated graphs presenting tumor volume calculated using the following formula: Length*Width^2^/2. In the updated graphs, the control groups exceed 2000 mm^3^. The corresponding author provided details about welfare checks and stated that they were performed initially three times per week, and daily when tumors became larger. They stated that in the final two weeks of the experiment, some mice lost weight. The size and condition of tumors, and the fact that some mice lost weight, call into question whether the humane endpoint criteria or monitoring frequency were sufficient to mitigate animal welfare concerns in the reported experiments.

The *PLOS ONE* Editors consulted with a member of the Editorial Board, who confirmed that tumor volumes reported in the updated graphs for Fig 6 exceed internationally-accepted standards. Concerns regarding tumor volume indicate that the article is not in compliance with the *PLOS* Animal Research policy which requires that, “studies involving animals must be conducted according to internationally-accepted standards.”

The corresponding author provided the following sequence for the control siRNA used in the siRNA knockdown experiment: 5’-TTCTCCGAACGTGTCACGT-3’.

For Fig 5A, the corresponding author stated that the KYSE150 Control 0 h panel was included in error. They provided a replacement panel, along with the raw data for this figure.

For western blot panels in Figs 2A and 4, the corresponding author confirmed that proteins were measured on different membranes. For Fig 4, they explained that this was due to difficulty probing for proteins of a similar size. They originally attempted to strip and re-probe the same membrane but were unsuccessful. For Figs 2C and S1A, the corresponding author stated that IQGAP1 and β-actin were measured on the same membranes, but different lanes were presented for each protein to improve presentation.

In light of the above concerns regarding animal welfare which indicate that the article is not in compliance with the *PLOS* Animal Research policy, the *PLOS ONE* Editors retract this article. The editors regret that the issues in this article were not identified prior to publication.

XXW agreed with the retraction, stands by the article’s findings, and apologizes for the issues with the published article. All other authors either did not respond directly or could not be reached.
